# Radiotherapy for patients with brain metastases and leptomeningeal carcinomatosis: prognostic factors and clinical outcomes

**DOI:** 10.1007/s10585-025-10352-3

**Published:** 2025-06-02

**Authors:** Lena Maria Blattmann, Rami El Shafie, Stephanie Bendrich, Sandra Donath, Olga Knaus, Andrea Hille, Tammam Abboud, Manuel Guhlich, Martin Leu, Markus Anton Schirmer, Mahalia Zoe Anczykowski, Laura Anna Fischer, Benedikt Kieslich, Philipp Jung, Stefan Rieken, Carla Marie Zwerenz, Leif Hendrik Dröge

**Affiliations:** 1 Department of Radiotherapy and Radiation Oncology, University Medical Center Göttingen, Robert-Koch-Str. 40, 37075 Göttingen, Germany; 2 Göttingen Comprehensive Cancer Center (G-CCC), University Medical Center Göttingen, Von-Bar-Str. 2/4, 37075 Göttingen, Germany; 3 Department of Neurosurgery, University Medical Center Göttingen, Robert-Koch-Str. 40, 37075 Göttingen, Germany

**Keywords:** Brain metastases, Leptomeningeal carcinomatosis, Radiotherapy WBRT, Boost

## Abstract

Brain metastases and leptomeningeal carcinomatosis (LC) are complications of advanced-stage malignancies, associated with a poor prognosis. This study aimed to evaluate the role of prognostic factors and radiotherapy (RT) treatment approaches while taking toxicity into account. We performed a retrospective study and compared clinical characteristics, prognostic factors, toxicities and outcomes in patients with (1) parenchymal brain metastases (PM) (*n* = 275) vs. LC (*n* = 35) and (2) in patients with whole brain radiotherapy (WBRT) (*n* = 52) vs. WBRT + boost (*n* = 201). We found poorer survival (OS) of the LC group compared to PM patients in univariable analysis (not in multivariable analysis). LC patients predominantly underwent WBRT only, received surgical resection before RT less frequently and had more RT discontinuations than PM patients. OS was better in the WBRT + boost group than in the WBRT only group. In patients who received WBRT + boost, the primary tumor was more often controlled, and the number of PM was lower compared to the WBRT only group. WBRT + boost was associated with higher rates of alopecia than WBRT only. Patients with LC had a worse prognosis compared to patients with PM. WBRT + boost resulted in higher toxicity than WBRT only but resulted in better OS in the presented study. WBRT + boost patients had more favorable prognostic factors prior to RT, so OS improvement is not likely due to boost. Treating brain metastases requires a careful assessment of benefits and risks. Optimal RT planning should consider prognostic factors and potential side effects individually.

**Supplementary Information:**

The online version contains supplementary material available at 10.1007/s10585-025-10352-3.

## Introduction

The occurrence of brain metastases and especially leptomeningeal carcinomatosis (LC) is a severe complication of advanced-stage malignancies and contributes significantly to the morbidity and mortality. Although present brain metastases are often not diagnosed because they are asymptomatic until patients’ death, one assumes that they occur in 25% of patients with malignant diseases [1]. Concerning the most frequent primary tumor, lung cancer is responsible for 50% of brain metastases. 15 to 20% of brain metastases appear from breast cancer, while gastrointestinal cancers, urogenital cancers and melanoma are the source of each 5 to 10% [2]. The incidence of LC in case of a malignant tumor is approximately 10% [3]. It is a result of the infiltration of the leptomeninges by malignant cells. While typical symptoms of brain metastases include nausea, headache and vomiting or hemiparesis, LC often also presents spinal manifestations such as sensory disorders or back pain [3–5].

Radiotherapy remains a cornerstone in the management of brain metastases and LC. Technological advancements, including stereotactic radiosurgery (SRS) and whole brain radiotherapy plus an integrated boost (WBRT + boost), have improved the precision and efficacy of treatment, minimizing damage to healthy tissue while targeting malignant cells [6]. Patients with a limited amount of solid brain metastases often benefit from locoregional treatment like surgical resection and SRS. In contrast, WBRT is a common option for patients with multiple solid brain metastases and especially LC [2]. When combining whole brain radiotherapy (WBRT) with a simultaneous integrated boost, studies found evidence for a survival benefit [7, 8].

However, the optimal radiotherapy strategy to manage these complex conditions is still a subject of ongoing research, particularly in the context of improving survival outcomes and quality of life while balancing treatment-related toxicities. The current study aims to explore the current role of radiotherapy in the treatment of brain metastases and LC. Specifically, this study intends to present a two-part-analysis, including a comparison of clinical characteristics, prognostic factors, toxicities and outcomes in patients with PM vs. LC and in patients treated with WBRT only vs. WBRT + boost.

## Materials and methods

### Study design

**Fig. 1:**
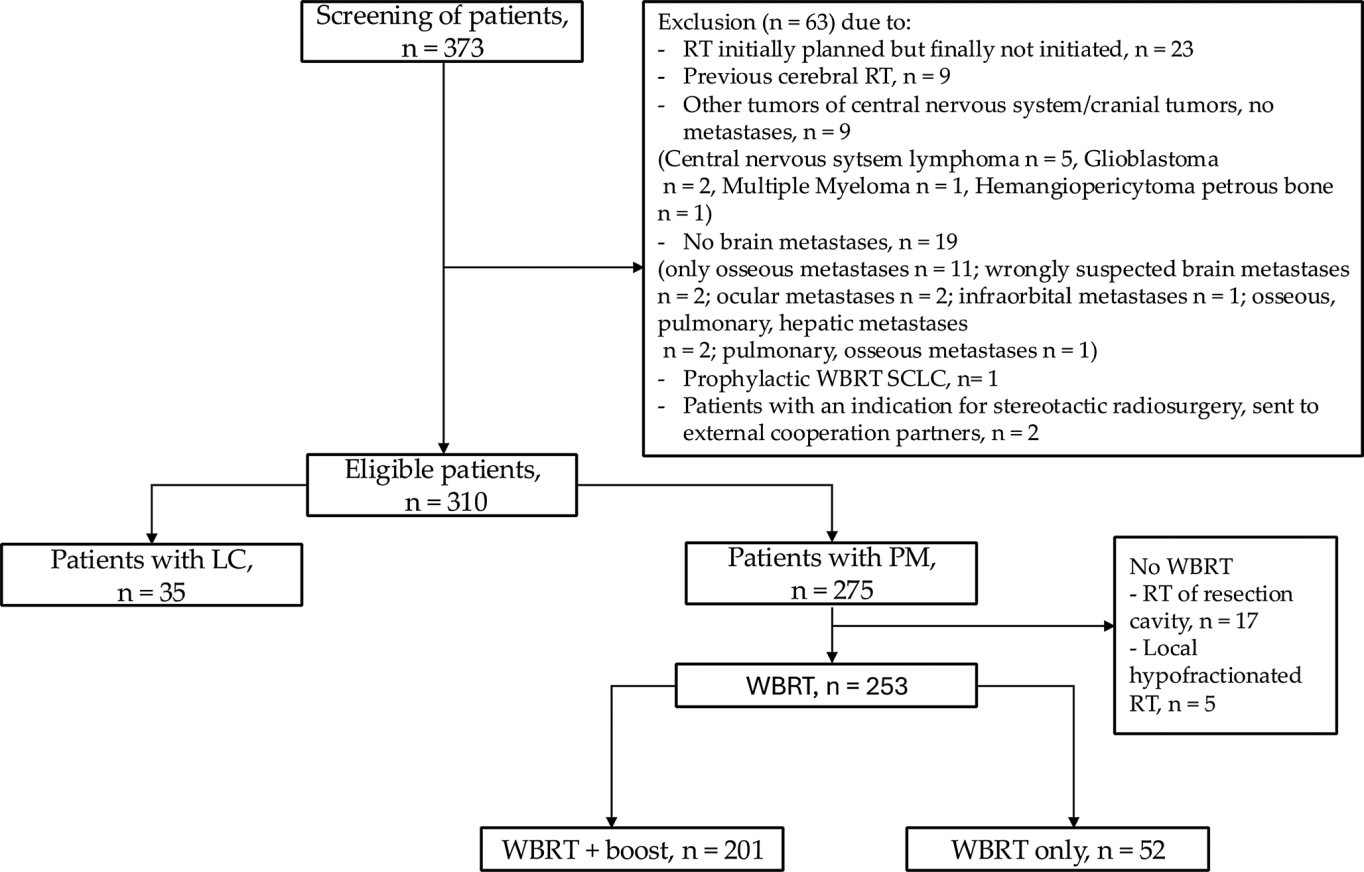
Flow chart. RT—radiotherapy. WBRT—whole brain radiotherapy. LC—leptomeningeal carcinomatosis. PM—parenchymal metastases. SCLC—small-cell lung cancer

The study was approved by the Institutional Ethics Committee of the University Medical Center of Göttingen (protocol code 14/12/23, date of approval 04/12/23). Patients with brain metastases and/or LC, who had undergone any RT at the University Medical Center from July 2015 to December 2023, were screened retrospectively. 373 patients were identified. In consideration of the exclusion criteria, finally 310 patients were eligible for the presented study. Exclusion criteria were for example a previous cerebral RT, the diagnosis of other tumors of central nervous system or cranial tumors (no metastases) and patients who had an indication for SRS. Further exclusion criteria are listed in Fig. 1.

For the first subproject of our study, we compared patients with parenchymal brain metastases (PM) (*n* = 275) vs. patients with LC (*n* = 35), to focus on the predominant disease without comparing the RT concepts.

As the second subproject, we compared two different RT concepts: WBRT only vs. WBRT + boost (Fig. 1, right part): First, we only included the PM patients without LC (*n* = 275), in order to have comparable baseline conditions. Next, patients who had not undergone WBRT (only local RT or SRS) were excluded; the reason was an expected bias – especially regarding the endpoint ICPFS and RT-related toxicity. From the remaining 253 patients, we finally formed the two subgroups: 201 patients who received WBRT + boost vs. 52 patients treated with WBRT only.

### Treatment

In the presented study, patients had undergone RT at the University Medical Center Göttingen. The neuro-oncological center is part of the University Cancer Center (G-CCC) and is certified since 2021 by the German Cancer Society (Deutsche Krebsgesellschaft, DKG, Berlin, Germany) [9]. To confirm the diagnosis of brain metastases, every patient had to undergo an imaging of the brain. Preferably, it would have been a magnetic resonance imaging (MRI), unless there existed medical contraindications e.g. a pacemaker, so patients received a computer tomography (CT) instead. The diagnosis of LC was based on either MRI and/or on positive cytology findings in cerebrospinal fluid. Every patient was discussed in the multidisciplinary tumor board. Therapy concepts and processes were based on national and international guidelines and SOPs (standard operation procedures) [10].

In the first step, patients received planning CT scans. The software Eclipse (Varian Medical Systems, Palo Alto, CA, USA) was used for RT planning. Varian linear accelerators with photon energies of 6 MeV or 20 MeV were utilized to apply the RT. To secure image guidance, we used electronic portal imaging device (EPID), on-board-kV-imaging (OBI), and cone-beam-CT (CBCT). Each patient received an individual thermoplastic mask. Either volumetric modulated arc therapy (VMAT, standard for WBRT + boost) or 3D conformal radiation therapy (3D-CRT, standard for WBRT only) were used. During the study´s observation period, due to technical conditions, stereotactic radiosurgery was not an available option at University Medical Center in Göttingen. When this kind of treatment was medically indicated, patients were referred to external cooperation partners. In this case, patients were excluded from the study (Fig. 1). The RT concept was chosen individually for each patient by the treating radiation oncologist. Whether WBRT only or WBRT + boost was used, was dependent on e.g. a limited number of PM or the existence of clinical symptoms. The decision making followed guidelines of the American Society for Radiation Oncology (ASTRO) [11]. In the case of WBRT only, a total dose of 30 Gy in 10 fractions was routinely applied. For WBRT + boost, the standard was WBRT with 12 fractions up to 30 Gy, along with a simultaneous boost to 45 Gy for metastases and resection cavities. Additionally, in cases with proximity to organs at risk, a total of 15 fractions were applied: These included 12 fractions of WBRT with a single dose of 2.5 Gy, while metastases or resection cavities simultaneously received a boost in 12 fractions with a single dose of 3 Gy, followed by a sequential boost of 3 fractions with a single dose of 3 Gy. Standard follow-up imaging included MRI scans quarterly for two years and then semi-annually or whenever new symptoms occurred. Follow-up was performed together with cooperation partners like primary tumor treating oncologists. The RT aftercare specifically focused on the evaluation of side effects and toxicity.

### Prognostic scores

To assign patients to homogenic groups regarding their prognosis, it was made use of prognostic scores of the Radiation Therapy Oncology Group (RTOG). The first score was the Recursive Partitioning Analysis (RPA) [12], followed by the Graded Prognostic Assessment (GPA) [13]. To specifically differentiate between the five most common primary tumors (lung cancer, melanoma, breast cancer, renal cell carcinoma, gastrointestinal cancers), we utilized the diagnose-specific Graded Prognostic Assessment (ds-GPA) [14]. During the past decade the RTOG published an updated version of each ds-GPA, which included, among other factors, genetic features e.g. mutations [15–19].

### Endpoints and statistical analysis

The toxicities were scored in accordance with the Common Terminology Criteria for Adverse Events (v5.0) [20]; the focus was on cranial-related toxicities. In clinical practice, the RANO-criteria (Response assessment in Neuro-Oncology) served as standard for response assessment [21]. The survival endpoints were overall survival (OS), intracranial progression-free survival (ICPFS) and freedom from cranial progression (FFCP). For OS only patient death was registered as an event. Regarding ICPFS, registered events were patient death or intracranial recurrence, while for FFCP, only intracranial recurrence counted as an event. Survival times were calculated from the last day of RT. Intracranial progress during RT was also considered; in some cases, this resulted in negative values for ICPFS. For data administration and descriptive statistics (Pearson’s chi-squared test, Mann-Whitney U test, Cox regression analysis, univariable and multivariable), we utilized the programs Statistica (v13.3, TIBCO Software Inc., Palo Alto, California, USA), Microsoft Excel (v2016, Microsoft Corporation, Redmond, Washington, DC, USA), and SPSS (v27, IBM Corp., Armonk, NY, USA). Additionally, the Software R (v4.1.0) with the plugin KMWin (v1.53) was used for illustration of survival curves [22]. As there here presented analyses are deemed exploratory, i.e. not corrected for multiplicity testing, a nominal p-value of < 0.05 was considered statistically significant. To test differences in survival, we used the logrank test and the Kaplan-Meier estimator. All parameters that showed significance (*p* < 0.05) in univariable analysis were included in multivariable models, which were run both in a conditional stepwise forward fashion (“stepwise”) and in a mode where all parameters were included at one step (“enter mode”). This was employed separately for each of the considered endpoints. Here, we only present the results of “enter mode”, since “stepwise” delivered almost identical results. In case of missing data due to retrospective research, those patients were not included in the affected analysis. Each case is documented in the belonging head of the table.

## Results

### Comparison of PM vs. LC

#### Clinical and treatment characteristics

Concerning age, sex, Karnofsky Performance Scale (KPS), Charlson Comorbidity Index (CCI) and primary tumors, we did not find any significant differences between the two groups. Please, see Table 1.

**Table 1:** Comparison of baseline and clinical characteristics in patients with LC vs. patients with PM. Median (minimum-maximum) values or numbers of patients (percentage) are presented, if not otherwise specified. ^1^Pearson’s chi-squared test, ^2^Mann-Whitney U test, ^3^further primary tumors: thyroid carcinoma *n* = 1, pleura mesothelioma *n* = 1, pharyngeal carcinoma *n* = 1; unavailable data due to retrospective research at: ^4^*n* = 2 patients, ^5^*n* = 12 patients, ^6^*n* = 14 patients. LC—leptomeningeal carcinomatosis. PM—parenchymal metastases. RT—radiotherapy. CUP—cancer of unknown primary. NSCLC—non-small-cell lung cancer. SCLC—small-cell lung cancer

Parameter	Patients with LC, *n* = 35	Patients with PM, *n* = 275	*p*-value
Age (years, median (min-max))	62 (20–80)	65.0 (16–91)	0.262^2^
Gender			0.101^1^
Male	15 (42.9)	158 (57.5)	
Female	20 (57.1)	117 (42.5)	
Karnofsky Index	80 (40–100)	80 (20–100)	0.325^2^
(median (min-max)			
Charlson Comorbidity Index (median (min-max))	8 (6–11)	9 (6–15)	0.134^2^
Primary tumor			0.545^1^
Bronchial carcinoma	16 (45.7)	154 (56.0)	
Melanoma	3 (8.6)	25 (9.1)	
Breast cancer	5 (14.3)	16 (5.8)	
Gastrointestinal cancer	4 (11.4)	31 (11.3)	
Urogenital cancer	6 (17.1)	34 (12.4)	
CUP	1 (2.9)	12 (4.4)	
Other primary tumors^3^	0	3 (1.1)	
Histology bronchial carcinoma			0.609^1^
NSCLC	14 (40.0)	112 (40.7)	
SCLC	2 (5.7)	39 (14.2)	
Unknown histology	1 (2.9)	3 (1.1)	
Other primary tumors	18 (51.4)	121 (44.0)	
Stage T0-T2	7 (20.0)	79 (28.7)	0.077^2^
Stage T3-T4	13 (37.1)	120 (43.6)	
Unknown T-stage	15 (42.9)	76 (27.6)	
Stage N0-N1	10 (28.6)	101 (36.7)	0.193^2^
Stage N2-N3	12 (34.3)	101(36.7)	
Unknown N-stage	13 (37.1)	73 (26.5)	
Primary controlled pre RT	27 (77.1)	224 (81.5)	0.541^1^
Extracranial controlled pre RT	22 (62.9)	180 (65.5)	0.761^1^
Initially extracranial metastases	24 (68.6)	156 (56.7)	0.181^1^
Number of brain lesions^6^	5 (0-176)	3 (1-172)	0.217^2^
(median (min-max))			
Infratentorial involvement	19 (54.3)^4^	134 (48.7)^5^	0.473^2^
Brainstem involvement	5 (14.3)^4^	27 (9.8)^5^	0.394^1^

In terms of treatment characteristics, a metastasis resection before the begin of RT was more frequently performed in the PM group (45.8% vs. 20.0%, *p* = 0.004). Additionally, the groups differed significantly regarding the RT concept (*p* < 0.001): For LC patients, WBRT only was chosen more often (57.1%) as clinically indicated with a median dose of 30 Gy, while PM patients received WBRT + boost by the majority (73.1%) with a median dose of 45 Gy. Radiotherapy was not completed as planned more often in the LC group (28.6% vs. 14.9%, *p* = 0.04). Thus, 89.5% of PM patients received at least 80% of the planned RT dose, whereas this was only the case at 77.1% of LC patients (*p* = 0.034). Regarding the period between the diagnosis of LC/PM and the start of the RT treatment, PM group presented a shorter median interval of 0.92 months vs. 1.31 months for the LC group (*p* = 0.039). Please see Table 2 for further information.

**Table 2:** Comparison of treatment characteristics in patients with LC vs. patients with PM. Median (minimum-maximum) values or numbers of patients (percentage) are presented, if not otherwise specified. ^1^Pearson’s chi-squared test, ^2^Mann-Whitney U test, ^3^indivdually, at the patients’s request and with a reduced general condition, only local RT of the solid well-defined meningeal metastases; unavailable data due to retrospective research at ^4^*n* = 4 patients. LC—leptomeningeal carcinomatosis. PM—parenchymal metastases. RT—radiotherapy. WBRT—whole brain radiotherapy

Parameter	Patients with LC, *n* = 35	Patients with PM, *n* = 275	*p*-value
Surgical resection of PM pre RT	7 (20.0)	126 (45.8)	0.004^1^
RT concept			< 0.001^1^
WBRT alone	20 (57.1)	52 (18.9)	
WBRT + boost	14 (40.0)	201 (73.1)	
Hypofractionated RT of resection cavity	0 (0)	17 (6.2)	
Local RT without WBRT	1 (2.9)^3^	5 (1.8)	
Applicated total dose [Gy]	30 (3–45)	45 (3.75-45)	< 0.001^2^
Planned total dose [Gy]	30 (20–45)	45 (30-48.75)	< 0.001^2^
RT discontinuation	10 (28.6)	41 (14.9)	0.040^1^
At least 80% of planned total dose applicated	27 (77.1)	246 (89.5)	0.034^1^
Systemic therapy concomitant with RT	14 (40.0)	109 (39.6)	0.967^1^
*Kind of systemic therapy concomitant with RT*:			
Checkpoint inhibitor	6 (17.1)	54 (19.6)	0.725^1^
Tyrosine kinase inhibitor	2 (5.7)	16 (5.8)	0.980^1^
Hormone therapy	1 (2.9)	9 (3.3)	0.896^1^
Chemotherapy	6 (17.1)	51 (18.5)	0.840^1^
Systemic therapy 3 months pre/post RT	27 (77.1)	198 (72.0)	0.521^1^
Kind of systemic therapy 3 months pre/post RT			
Checkpoint inhibitor	10 (28.6)	98 (35.6)	0.409^1^
Tyrosine kinase inhibitor	4 (11.4)	27 (9.8)	0.765^1^
Hormone therapy	4 (11.4)	13 (4.7)	0.101^1^
Chemotherapy	18 (51.4)	121 (44.0)	0.405^1^
Antiepileptics +/- 2 weeks	5 (14.3)	63 (22.9)	0.246^1^
pre/post RT			
Corticosteroids concomitant with or 3 months past RT	25 (71.4)	149 (54.2)	0.053^1^
cMRI pre RT	34 (97.1)	256 (93.1)	0.358^1^
cMRI post RT	8 (22.9)	101 (36.7)	0.106^1^
Time between diagnosis of brain lesions & RT	1.31 (0.03–24.05)	0.92 (0.07–9.53)^4^	0.039^2^
[months] (median (min-max)			
Time between diagnosis of primary tumor & diagnosis of brain lesions	9.26 (0.0-135.95)	2.07 (0.0-363.07)	0.603^2^
[months] (median (min-max)			

#### Prognostic scores

Suppl. Table S1 shows the distribution of patients across the different classes of prognostic scores, comparing PM vs. LC patients. There was no statistically significant difference concerning the RPA. Regarding the GPA score, we observed a significant difference: A larger proportion of the LC group (62.9%) reached a GPA score of 0 to 1, which corresponds to the poorest prognosis. 43.3% of PM patients fell into this category (*p* = 0.013). Concerning the ds-GPA of 2012, as well as the more recent version, we did not find any significant difference. It must be noted that some patients had to be excluded and consequently could not be assigned to these two scores, e.g. due to primary tumors not represented in the ds-GPA, missing information on the number of PM, missing histology and/or laboratory values and the fact that two patients did not have solid brain metastases but only LC. For further explanations, please see the legend of Suppl. Table S1.

#### Toxicities

We did not observe any significant differences regrading RT-related toxicities. Further details concerning the comparison of toxicities ≥ I° in patients with LC vs. with PM are shown in Suppl. Table S2.

#### Outcomes

**Fig. 2:**
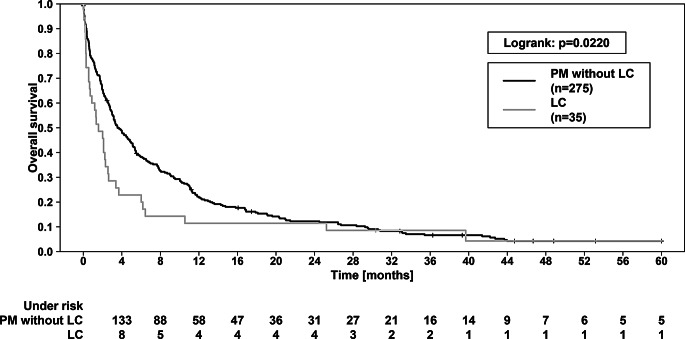
Overall survival (OS) of patients with LC vs. patients with PM. OS was significantly better in PM patients (*p* = 0.0220, logrank test). The 1-year and 2-year OS were 21.8% vs. 11.3% and 12.5% vs. 11.1%, respectively. LC—leptomeningeal carcinomatosis. PM—parenchymal metastases

**Fig. 3:**
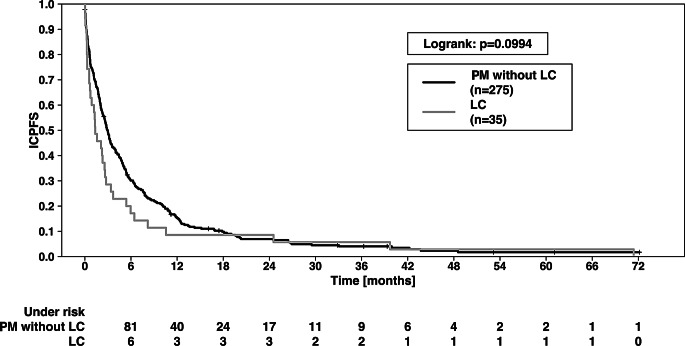
Intracranial progression free survival (ICPFS) of patients with LC vs. patients with PM. ICPFS showed a trend (*p* = 0.0994, logrank test). The 1-year and 2-year ICPFS were 15.2% vs. 8.6% and 6.9% vs. 8.2%, respectively. For graphic illustration, those 4 patients who showed intracranial progression already during RT treatment were set to “0”, to avoid negative values for ICPFS here, since survival was counted from the last day of RT. LC—leptomeningeal carcinomatosis. PM—parenchymal metastases

Concerning the overall study collective (*n* = 310), at the end of data evaluation, 292 patients were dead and 18 were alive. Cerebral progress after RT treatment was documented in 74 patients. In comparison of the PM- and LC group, there was a significant difference in OS (Fig. 2): the 1-year OS of PM and LC patients was 21.8% vs. 11.3%, while 2-year OS was 12.5% vs. 11.1% (*p* = 0.022 in logrank test). The median OS was 3.55 vs. 1.58 months (*p* = 0.004). Regarding ICPFS, there was a trend towards a superior outcome of the PM group (*p* = 0.0994; Fig. 3). We found no significant difference for FFCP (*p* = 0.822).

### Comparison of WBRT vs. WBRT + boost

#### Clinical and treatment characteristics

Clinical and treatment characteristics were compared between the WBRT- and WBRT + boost group. Before the start of RT, the primary tumor was more often controlled in the WBRT + boost group (85.1% vs. 65.4%, *p* = 0.001). Furthermore, extracranial metastases (ECM) were more commonly found in the WBRT only group (73.1% vs. 54.7%, *p* = 0.017). Another significant difference was observed regarding the number of brain metastases (*p* < 0.001): The median number was 3 in the WBRT + boost group, whereas it was 13.5 in patients who were treated with WBRT only. Additionally, infratentorial and brainstem involvement was more frequently represented in the WBRT only group (59.6% vs. 47.3%, *p* = 0.001 and 23.5% vs. 7.5%, *p* < 0.001). Please refer to Table 3 for further information.

**Table 3:** Comparison of baseline and clinical characteristics in patients with WBRT + boost vs. patients with WBRT only. Median (minimum-maximum) values or numbers of patients (percentage) are presented, if not otherwise specified. ^1^Pearson’s chi-squared test, ^2^Mann-Whitney U test, ^3^further primary tumors: thyroid carcinoma *n* = 1, pleura mesothelioma *n* = 1, pharyngeal carcinoma *n* = 1; unavailable data due to retrospective research at: ^4^*n* = 1 patient, ^5^*n* = 11 patients, ^6^*n* = 13 patients. WBRT—whole brain radiotherapy. RT—radiotherapy. CUP—cancer of unknown primary. NSCLC—non-small-cell lung cancer. SCLC—small-cell lung cancer

Parameter	WBRT + boost, *n* = 201	WBRT only, *n* = 52	*p*-value
Age (years, median (min-max))	65 (35–88)	64 (16–91)	0.366^2^
Gender			0.413^1^
Male	111 (55.2)	32 (61.5)	
Female	90 (44.8)	20 (38.5)	
Karnofsky Index	90 (20–100)	80 (40–100)	0.275^2^
(median (min-max))			
Charlson Comorbidity Index	9 (6–15)	8 (6–12)	0.345^2^
(median (min-max))			
Primary tumor			0.206^1^
Bronchial carcinoma	112 (55.7)	33 (63.5)	
Melanoma	19 (9.5)	1 (1.9)	
Breast cancer	10 (5.0)	6 (11.5)	
Gastrointestinal carcinoma	23 (11.4)	3 (5.8)	
Urogenital carcinoma	24 (11.9)	7 (13.5)	
CUP	10 (5.0)	2 (3.8)	
Other primary tumors^3^	3 (1.5)	0 (0)	
Histology bronchial carcinoma			< 0.001^1^
NSCLC	88 (43.8)	15 (28.8)	
SCLC	21 (10.4)	18 (34.6)	
Unknown histology	3 (1.5)	0 (0)	
Other primary tumors	89 (44.3)	19 (36.5)	
Stage T0-T2	60 (29.9)	14 (26.9)	0.931^2^
Stage T3-T4	83 (41.3)	24 (46.2)	
Unknown T-stage	58 (28.9)	14 (26.9)	
Stage N0-N1	77 (38.3)	16 (30.8)	0.538^2^
Stage N3-N4	69 (34.4)	22 (42.3)	
Unknown N-stage	55 (27.4)	14 (26.9)	
Primary controlled pre RT	171 (85.1)	34 (65.4)	0.001^1^
Extracranial controlled pre RT	132 (65.7)	33 (63.5)	0.766^1^
Initially extracranial metastases	110 (54.7)	38 (73.1)	0.017^1^
Number of brain lesions^6^	3 (1–90)	13.5 (1-172)	< 0.001^1^
(median (min-max))			
Infratentorial involvement	95 (47.3)^4^	31 (59.6)^5^	0.001^1^
Brainstem involvement	15 (7.5)^4^	12 (23.5)^5^	< 0.001^1^

Regarding treatment characteristics, WBRT + boost patients were more often treated with a surgical metastasis resection before the start of RT (49.3% vs. 19.2%, *p* < 0.001). We observed a trend (*p* = 0.072) concerning the number of patients who received at least one MRI scan after RT (WBRT only, 23.1%, WBRT + boost, 36.3%; Table 4).

**Table 4:** Comparison of treatment characteristics in patients with WBRT + boost vs. patients with WBRT only. Median (minimum-maximum) values or numbers of patients (percentage) are presented, if not otherwise specified. ^1^Pearson’s chi-squared test, ^2^Mann-Whitney U test. RT—radiotherapy. WBRT—whole brain radiotherapy

Parameter	WBRT + boost, *n* = 201	WBRT only, *n* = 52	*p*-value
Surgical resection of PM pre RT	99 (49.3)	10 (19.2)	< 0.001^1^
Applicated total dose [Gy]	45 (3.75-45)	30 (6–36)	< 0.001^2^
RT discontinuation	29 (14.4)	11 (21.2)	0.236^1^
At least 80% of planned total dose applicated	180 (89.6)	45 (86.5)	0.537^1^
Systemic therapy concomitant with RT	75 (37.3)	22 (42.3)	0.509^1^
*Kind of systemic therapy concomitant with RT*:			
Checkpoint inhibitor	35 (17.4)	9 (17.3)	0.986^1^
Tyrosine kinase inhibitor	14 (7.0)	1 (1.9)	0.170^1^
Hormone therapy	6 (3.0)	(3.8)	0.752^1^
Chemotherapy	33 (16.4)	13 (25.0)	0.153^1^
Systemic therapy 3 months pre/post RT	142 (70.6)	40 (76.9)	0.369^1^
Kind of systemic therapy 3 months pre/post RT			
Checkpoint inhibitor	67 (33.3)	17 (32.7)	0.930^1^
Tyrosine kinase inhibitor	22 (10.9)	4 (7.7)	0.491^1^
Hormone therapy	7 (3.5)	5 (9.6)	0.064^1^
Chemotherapy	85 (42.3)	29 (55.8)	0.082^1^
Antiepileptics +/- 2 weeks pre/post RT	47 (23.4)	11 (21.2)	0.733^1^
Corticosteroids concomitant with or 3 months post RT	119 (59.2)	25 (48.1)	0.149^1^
cMRI pre RT	189 (94.0)	45 (86.5)	0.068^1^
cMRI post RT	73 (36.3)	12 (23.1)	0.072^1^
Time between diagnosis of brain lesions & RT [months] (median (min-max)	1.30 (0.30-24.05)	1.12 (0.03–19.02)	0.638^2^

#### Prognostic scores

The distribution of patients across the RPA classes did not show any significant differences. Concerning the GPA score, a significant majority of the WBRT only group (65.4%) reached a GPA-score of 0 to 1 compared to 41.8% of the WBRT + boost group (*p* < 0.001). In the more recent version of the ds-GPA, most patients treated with WBRT + boost had scores of 1.5 to 2 and 2.5 to 3 (combined 53.2%), while it was only 30.8% in the group without boost (*p* = 0.029). As explained above for the comparison of PM vs. LC, some patients also had to be excluded from the analysis in the comparison of WBRT vs. WBRT + boost. For more details, please refer to Suppl. Table S3.

#### Toxicities

The distribution of cranial RT treatment-related side effects in comparison of the two subgroups is shown in Suppl. Table S4. There was no significant difference between the two RT concepts, when stratified for any cranial side effects or for the highest grade of observed toxicity. Regarding single toxicity items, alopecia of grade 1 or higher occurred significantly more often in the WBRT + boost group (10.4%), while none of the patients in the WBRT only group were affected (*p* = 0.015).

#### Outcomes

**Fig. 4:**
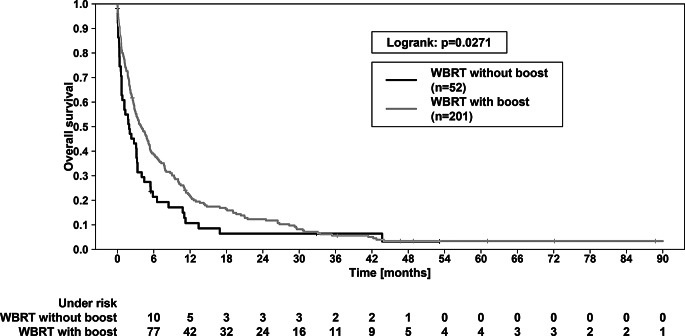
Overall survival (OS) of patients treated with WBRT + boost vs. patients treated with WBRT only. OS was significantly better in PM patients (*p* = 0.0271, logrank test). The 1-year and 2-year OS were 21.6% vs. 10.5% and 11.8% vs. 5.9%, respectively. WBRT—whole brain radiotherapy

**Fig. 5:**
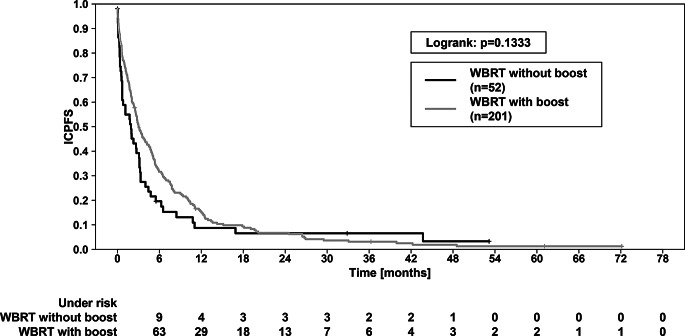
Intracranial progression free survival (ICPFS) of patients treated with WBRT + boost vs. patients treated with WBRT only. ICPFS showed a trend (*p* = 0.1333, logrank test). The 1-year and 2-year ICPFS were 15.0% vs. 8.2% and 6.9% vs. 6.2%, respectively. For graphic illustration, those 4 patients who showed intracranial progression already during RT treatment were set to “0”, to avoid negative values for ICPFS here, since survival was counted from the last day of RT. WBRT—whole brain radiotherapy

In comparison the two RT concepts, a significant survival advantage in terms of OS was observed for the WBRT + boost group (*p* = 0.0271 in logrank test; Fig. 4). The 1-year-OS for the boost group was 21.6% compared to 10.5% for the WBRT only group. The 2-year-OS was 11.8% vs. 5.9%. The median OS was 3.84 vs. 1.87 months (*p* < 0.001). Regarding ICPFS, there was no significant difference (*p* = 0.1333; Fig. 5). FFCP also did not show any significant difference between the WBRT + boost vs. WBRT only group (*p* = 0.185).

#### Prognostic factors (WBRT only vs. WBRT + boost patients)

To analyze prognostic factors in PM-patients treated with WBRT (WBRT only and WBRT + boost, *n* = 253), first, we performed univariable analysis (Suppl. Table S5). Here, LC patients were excluded from the analysis. In univariable analysis, the following parameters showed a significant effect (*p* < 0.05) on at least one of the survival endpoints: gender, age, KPS, CCI, number of brain metastases (< 5 vs. ≥ 5), primary tumor control before RT, the presence of ECM at the time of diagnosis of PM, surgical resection, WBRT + boost, infratentorial involvement, total dose applied, RT side effects and a therapy with corticosteroids concomitant with or within 3 months after RT, planned total dose and cMRI post RT. In the multivariable analysis, age, KPS, primary tumor control before RT, RT side effects, surgical resection, total dose applied, and an additional RT boost had a significant influence on OS. Concerning ICPFS, gender, KPS, age, primary control pre RT, RT side effects and a therapy with corticosteroids concomitant with or within 3 months after RT were significant prognostic factors, in multivariable analysis. Further details of the multivariable analysis of prognostic factors for the WBRT groups are shown in Suppl. Table S6.

### General prognostic factors (overall study collective)

For the overall collective of the present study (*n* = 310), we pointed out prognostic factors by performing a univariable cox regression analysis (Suppl. Table S7). The following parameters showed a significant effect (*p* < 0.05) on at least one of the survival endpoints: age, KPS, CCI, number of brain metastases (< 5 vs. ≥ 5), primary tumor control before RT, the presence of ECM at the time of diagnosis of PM, the presence of LC, previous metastasis resection, RT side effects, WBRT, total dose applied, a systemic therapy within 3 months before or 3 months after RT, chemotherapy mono vs. targeted therapy/immune therapy (+/− chemotherapy) 3 months before or after RT, infratentorial involvement, a therapy with corticosteroids concomitant with within or 3 months after RT and the period of time between the diagnosis of PM/LC and the start of RT treatment. Table 5 provides the results of the multivariable analysis for the entire study cohort (*n* = 310). Age, KPS, WBRT, RT side effects, total dose applied and a therapy with steroids concomitant with or 3 months post RT had a significant influence on OS. KPS, primary control pre RT, RT side effects, total dose applied and a therapy with steroids concomitant with or 3 months post RT were significant prognostic factors regarding ICPFS. While LC as a prognostic factor still showed a significant influence on survival in the univariable analysis, it was lost in multivariable analysis.

**Table 5:** Prognostic factors in overall study collective, multivariable Cox regression analysis. In univariable analysis, the endpoint “FFCP” was associated with a p-value < 0.05 for “charlson comorbidity index,” in addition to “cmri post RT,” which strongly reflects the outcome and is thus considered as a bias. Therefore, it was not included in the multivariable analysis, and a multivariable model was not conducted at this point. The impact of one variable, namely “systemic therapy 3 months before or after RT,” identified in the univariable analysis with *p* = 0.03 in relation to OS, was excluded by the software from the multivariable model. This occurred because the degree of freedom for this variable was reduced to zero, either due to a constant or linearly dependent covariates, making it impossible to calculate an estimator. Unavailable data due to retrospective research at: ^1^*n* = 15 patients, ^2^*n* = 14 patients, ^3^*n* = 85 patients did not receive any systemic therapy 3 months before or after RT. OS—overall survival. ICPFS—intracranial progression-free survival. FFCP—freedom from cranial progression. HR—hazard ratio. CI—confidence interval. RT—radiotherapy. KPS—Karnofsky performance status scale. CCI—Charlson comorbidity index

Parameter (Number of patients)	OS		ICPFS	
	HR (95% CI)	*p*-value	HR (95% CI)	*p*-value
Age at RT	1.03 (1.01–1.04)	**< 0.001**	-	-
Karnofsky Index ≤median (159) vs. > median (151) Median: 80	1.99 (1.46–2.72)	**< 0.001**	1.77 (1.37–2.28)	**< 0.001**
Number of brain lesions^1^ ≥ 5 (126) vs. <5 (169)	1.14 (0.78–1.67)	0.51	1.05 (0.76–1.45)	0.776
Primary controlled pre RT No (59) vs. Yes (251)	1.34 (0.93–1.93)	0.119	1.42 (1.03–1.95)	**0.030**
Extracranial metastases at brain lesion diagnosis Yes (180) vs. No (130)	1.09 (0.79–1.50)	0.606	1.00 (0.77–1.30)	0.993
Leptomeningeal carcinomatosis Yes (35) vs. No (275)	1.22 (0.78–1.91)	0.380	-	-
Surgical resection No (177) vs. Yes (133)	1.04 (0.75–1.45)	0.818	0.77 (0.58–1.02)	0.068
WBRT Yes (287) vs. No (23)	2.33 (1.22–4.46)	**0.011**	-	-
RT side effects Yes (122) vs. No (188)	0.68 (0.50–0.92)	**0.012**	0.75 (0.58–0.97)	**0.029**
Total dose applied ≤ 30 Gy (109) vs. >30 Gy (201)	1.98 (1.42–2.76)	**< 0.001**	1.60 (1.22–2.10)	**0.001**
At least 1 lesion in cerebellum^2^ Yes (153) vs. No (143)	1.06 (0.74–1.51)	0.766	1.26 (0.94–1.71)	0.127
Steroids concomitant with or 3 months post RT Yes (174) vs. No (136)	1.37 (1.00-1.87)	**0.048**	1.52 (1.18–1.97)	**0.001**
Chemotherapy mono (84) vs. targeted therapy/immune therapy +/− chemotherapy (141) 3 months before or after RT^3^	1.26 (0.93–1.73)	0.140	-	-

## Discussion

The development of brain metastases or LC remains one of the most unfavorable prognostic events [23, 24]. Although especially LC is diagnosed in 1 to 8% of cancer patients [25], there is still only insufficient evidence on therapeutic approaches [26]. Additionally, regarding the RT of brain metastases, it is often discussed whether an additional boost to WBRT brings relevant prognostic benefits, or just significantly increases toxicity [27]. In the current study, we compared clinical and treatment related characteristics, toxicity and outcomes of patients with PM vs. LC. In addition, we compared WBRT only vs. WBRT + boost concerning the same aspects.

We found a significant disadvantage in survival for LC patients in comparison to PM patients (1-year-OS: 11.7% vs. 23,7%). Li et al. compared 185 PM patients with 102 LC patients; all of them were non-small cell lung cancer (NSCLC) patients. In line with our findings, they reported a superior median OS for the PM group [28]. The difference in survival could be explained by a poorer general condition and the presence of more comorbidities of the LC patients in advance. This assumption can be made regarding the different distribution of patients to the RTOG prognostic scores in our study. Here, we found that the vast majority of LC patients were assigned to a GPA score with poorer prognosis. Multiple further studies have highlighted the substantial prognostic impact of the performance status, especially in LC patients [29–31]. Alongside this, Li et al. reported a lower performance status in the LC group than in the PM group [28]. The difference could be attributed to the fact that LC exhibits a biological behavior with higher malignancy. At the time when LC firstly occurs, the tumor disease appears to be already in a very advanced stage, which strongly impacts the patient’s performance status. This can be assumed because, in comparison, 68.6% of LC patients in proportion to only 56.7% of PM patients presented ECM at the time of diagnosis of LC/PM in the present study, which did not show statistical significance (*p* = 0.181). Nevertheless, while LC was a significant prognostic factor in univariable analysis, it was lost in multivariable analysis in the present study. This could be possibly explained by the smaller cohort of LC patients (*n* = 35) in proportion to the PM cohort (*n* = 275). Zhang et al. reported that LC did not show significance regarding OS in univariable analysis in their study [32]. But it must be noted that they presented an even smaller case number of LC (8 vs. 165 PM) patients, because it was not in the study´s focus. In addition, in the present study it was demonstrated that treatment discontinuation during ongoing RT occurred significantly more often in the LC group. This observation can be attributed to the poorer general condition and performance status, as previously discussed.

In the second part of our study, the two RT concepts WBRT vs. WBRT + boost were compared. We found a significant OS advantage of the WBRT + boost group compared to the WBRT only group (median 4.47 months vs. 2.32 months from the beginning of RT). Sun et al. reported similar outcomes while comparing 33 patients who underwent WBRT + boost vs. 49 patients who received WBRT only, all patients had small cell lung cancer (SCLC). They also found significant differences in median OS (13.4 vs. 8.5 months, *p* = 0.004) [7]. In the present study, the difference could be explained by a poorer prognosis of the WBRT only patients in advance, before the beginning of RT treatment. This assumption can be made because significantly more WBRT only patients were assigned to a lower GPA score. This was the same for the updated version of ds-GPA. Zhang et al. found comparable differences, 69.6% of WBRT + boost patients vs. 51.8% of WBRT only patients were allocated to a GPA ≥ 2 which means a more favorable prognosis [32]. This was in line with findings by Dobi et al. regarding the RPA classes [33].

In multivariable analysis, an additional boost to WBRT was a relevant prognostic factor concerning OS. Also previous studies reported that the RT concept had a significantly independent effect on OS [34, 35]. However, in their multivariable analysis, Zhang et al. were able to demonstrate that the RT concept of WBRT + boost compared to WBRT alone significantly improved ICPFS in the affected patients as an independent prognostic factor (*p* = 0.026), but not OS [32]. In the presented study, there was no difference in terms of ICPFS between WBRT + boost and WBRT only patients. Combined with the above finding – that the improved OS of WBRT + boost patients might be not likely due to boost but rather due to a better functional status – it can be stated that a boost should be considered carefully.

Regarding treatment-related characteristics, in a significantly higher proportion of the WBRT + boost group, surgical resection had already been performed prior to RT. Dobi et al. observed a similar distribution in their study, whereat 49.6% of WBRT + boost patients and only 9.7% of the WBRT group had undergone surgical resection before RT [33]. A previous resection is considered as an indication for WBRT + boost, allowing increased dose to the resection cavity in the adjuvant setting to reduce recurrence rates. Rades et al. investigated this issue, analyzing the outcomes of 201 patients who had one to two brain metastases resected, followed by either WBRT + boost (102 patients) or WBRT alone (99 patients). Patients who received WBRT + boost after the resection had a significantly better OS [36].

In the present study, WBRT + boost patients experienced more alopecia (≥ grade 1). While alopecia occurred in 10.4% of WBRT + boost group, it was not documented in a single case of the WBRT only group. A high incidence of alopecia regarding WBRT + boost was also reported by Popp et al. It occurred in all 62 patients of their WBRT + boost cohort [8]. In a prospective study of Gerrard et al., all patients treated with WBRT experienced alopecia [37]. Compared to the findings of Gerrard et al., the alopecia rate is very low in the presented study. One possible explanation could be the retrospective nature of the presented study and the short follow-up period of these patients in a palliative care setting. Though, Münstedt et al. found evidence that cancer therapy-induced alopecia, mostly studied regarding chemotherapy-induced alopecia, can cause changes to the self-concept and body image [38]. It is often ranked as one of the most distressing adverse effects and has an impact on the quality of life [39–41]. Thus, it can be stated that alopecia as a result of RT-treatment can lead to relevant impairment for the affected patients.

Additionally, in terms of prognostic factors, the multivariable analysis (WBRT cohort) in the present study showed that gender had a significant influence on ICPFS, but not on OS. Moreover, it was demonstrated that female gender exhibited a prognostic advantage in terms of ICPFS. This was in line with findings by Gupta et al. who studied prognostic factors in 116 patients with brain metastases and reported a significant superior survival for female compared to male patients (*p* = 0.015) [42]. In contrast, Sperduto et al. conducted a multicenter study (RTOG) with large cohorts in which they did not find any gender specific differences in OS [43]. In contrast, Smedby et al. found that women exhibited a longer median survival [44]. However, reasons for the observed differences cannot be explained by the presented study; further investigations would be necessary to clarify.

Furthermore, we found KPS to have a significant influence on OS and ICPFS. Freeman et al. similarly investigated a significant HR of 2.95 for OS and 1.86 for PFS regarding KPS ≤ 60 vs. > 60 [45]. Concludingly, it can be stated that a higher KPS is associated with a better prognosis. In the present study, primary control had a significant impact on ICPFS, while significance concerning OS was lost in multivariable analysis. Gaspar et al. previously reported a prognostic advantage in patients with a controlled primary disease, thus it was included in the RPA-score [12]. In univariable analysis (but not in multivariable analysis), another significant factor for OS was the presence of ECM in the present study which is in line with findings by other studies [32, 34]. Regarding the localization of metastases in the brain, infratentorial involvement appeared to have a poorer prognosis in the present study (only in univariable, not in multivariable analysis). Heimann et al. even reported an increased risk of death of 58% (*p* = 0.011, HR = 1.58) [46]. An explanation could be that infratentorial involvement implies a higher rate of complications. Steinruecke et al. studied that neurological and non-neurological post-operative complications were more frequent in patients with infratentorial metastases when comparing to patients with supratentorial metastases (neurological 21% vs. 13%, non-neurological 25% vs. 2%, *p* = 0.002) [47].

The present study had several limitations. Due to the retrospective nature of this study, all data were based on medical records. Thus, some data were incomplete e.g. the number of metastases in 14 patients whose pre-RT MRI scans were not available. It should also be mentioned that WBRT nowadays is used less for the treatment of brain metastases, as the focus is gradually shifting towards more effective precision RT options. This includes, for example, stereotactic radiosurgery. Additionally, the study results may be influenced by the described differences in baseline characteristics within the group as well as the distribution of the studied diagnosis. A bias due to not adjusting for parameters like performance status prior to RT or functional comorbidities should be considered. In the context of WBRT-related toxicity, another limitation is the missing consideration of cognitive or functional outcomes. It is the same explanation for the comparably low alopecia rate: This results from retrospective data research and a short follow-up period. Nevertheless, the current study provides a detailed analysis of real-world-data concerning prognostic factors and clinical outcomes of patients with brain metastases and LC treated with WBRT +/- boost, it addresses relevant questions in the management of these patients and therefore may contribute to a management. Especially regarding LC, minimal data exists on the affected patients. The current study may stimulate further research, specifically to study this topic in multicenter registry studies.

## Conclusion

During past decades, relevant progress has been achieved in the multimodal treatment of brain metastases. Nevertheless, the identification of the optimal treatment strategy is challenging in patients with brain metastases and especially LC. Here, we present a retrospective single-center study on outcome, prognosis, clinical characteristics and toxicity (1) in patients with PM vs. LC and (2) in patients with WBRT alone vs. WBRT + boost. We found poorer outcome in LC patients but no independent effect on survival in multivariable analysis, which could be attributed to the fact that those had a poorer prognosis and general condition in advance, prior to RT. Taken together, LC has indeed a poorer prognosis, but there is evidence that other prognostic factors like gender, KPS and primary tumor control also should be strongly considered in clinical decision making. When comparing RT concepts, patients who underwent WBRT + boost showed improved survival compared with those who received WBRT alone. This prognostic effect concerning OS could also be confirmed in multivariable analysis. Nevertheless, it must be emphasized that WBRT + boost patients had a more favorable functional status prior to RT, so it can be stated that OS improvement is not likely due to boost. Additionally, WBRT + boost was associated with higher rates of toxicities than WBRT alone. The limitations of this study regarding retrospective design, a treatment selection bias and the underpowered LC subgroup should be considered. In conclusion, it requires a critical evaluation of benefits and risks when determining the RT-indication. The planning of an optimal radiation oncology concept for patients with brain metastases should include a consideration of prognostic factors and potential side effects on an individual basis.

## Electronic supplementary material

Below is the link to the electronic supplementary material.

Supplementary Material 1

Supplementary Material 2

Supplementary Material 3

Supplementary Material 4

Supplementary Material 5

Supplementary Material 6

Supplementary Material 7

## Data Availability

The data presented in this study are available on reasonable request from the corresponding author.
