# Supplementary material for: Radiotherapy for patients with brain metastases and leptomeningeal carcinomatosis: prognostic factors and clinical outcomes
Source: Clin Exp Metastasis. 2025 Jun 2;42(4):33. doi: 10.1007/s10585-025-10352-3 (PMC12130157; doi:10.1007/s10585-025-10352-3)
Supplement: Supplementary file 1 — Supplementary Material 1 [file 10585_2025_10352_MOESM1_ESM.docx]

**Suppl Table S1:** Comparison of the distribution of patients with LC vs. patients with PM according to prognostic scores (RTOG). Numbers of patients (percentage) are presented, if not otherwise specified. Here, Mann-Whitney U test was used. The reasons for exclusion were as follows: ^1^n = 2 patients without PM, only LC; ^2^n = 1 patient with a missing number of brain metastases; ^3^n = 1 patient with CUP, n = 5 patients with primary tumors not considered in the score, n = 1 patient with a missing histological subtype in breast cancer, n = 2 patients with a missing number of brain metastases; ^4^n = 12 patients with CUP, n = 10 patients with a missing number of brain metastases, n = 1 patient with a missing histological subtype in breast cancer, n = 18 patients with primary tumors not considered in the score; ^5^n = 1 patient with CUP, n = 4 patients with a missing number of brain metastases, n = 1 patient with a missing histological subtype in breast cancer, n = 1 patient due to a missing hemoglobin value, n = 4 patients with primary tumors not considered in the score; ^6^n = 12 patients with CUP, n = 3 patients with missing histology in bronchial carcinoma, n = 11 patients with a missing number of brain metastases, n = 1 patient with a missing histological subtype in breast cancer, n = 18 patients with primary tumors not considered in the score, n = 6 patients with a missing hemoglobin value. LC—leptomeningeal carcinomatosis. PM—parenchymal metastases. RPA—Recursive Partitioning Analysis. GPA—Graded Prognostic Assessment. Ds-GPA—diagnosis-specific Graded Prognostic Assessment.

| **Parameter** | **Patients with LC, n = 35** | **Patients with PM, n = 275** | **p-value** |
| --- | --- | --- | --- |
| RPA |  |  | 0.747 |
| class 1 | 3 (8.6) | 28 (10.2) |  |
| class 2 | 27 (77.1) | 211 (76.7) |  |
| class 3 | 5 (14.3) | 36 (13.1) |  |
|  |  |  |  |
| GPA |  |  | 0.013 |
| 0 to 1 | 22 (62.9) | 119 (43.3) |  |
| 1.5 to 2 | 8 (22.9) | 106 (38.5) |  |
| 2.5 to 3 | 3 (8.6) | 43 (15.6) |  |
| 3.5 to 4 | 0 (0.0) | 6 (2.2) |  |
| Not classifiable | 2 (5.7)^1^ | 1 (0.4)^2^ |  |
|  |  |  |  |
| Ds-GPA (2012) |  |  | 0.100 |
| 0 to 1 | 12 (34.3) | 80 (29.1) |  |
| 1.5 to 2 | 11 (31.4) | 85 (30.9) |  |
| 2.5 to 3 | 1 (2.9) | 56 (20.4) |  |
| 3.5 to 4 | 2 (5.7) | 13 (4.7) |  |
| Not classifiable | 9 (25.7)^3^ | 41 (14.9)^4^ |  |
|  |  |  |  |
| Ds-GPA (updated version) |  |  | 0.061 |
| 0 to 1 | 7 (20.0) | 50 (18.2) |  |
| 1.5 to 2 | 13 (37.1) | 84 (30.5) |  |
| 2.5 to 3 | 3 (8.6) | 71 (25.8) |  |
| 3.5 to 4 | 1 (2.9) | 19 (6.9) |  |
| Not classifiable | 11 (31.4)^5^ | 51 (18.5)^6^ |  |
