# Supplementary material for: Radiotherapy for patients with brain metastases and leptomeningeal carcinomatosis: prognostic factors and clinical outcomes
Source: Clin Exp Metastasis. 2025 Jun 2;42(4):33. doi: 10.1007/s10585-025-10352-3 (PMC12130157; doi:10.1007/s10585-025-10352-3)
Supplement: Supplementary file 3 — Supplementary Material 3 [file 10585_2025_10352_MOESM3_ESM.docx]

**Suppl. Table S3:** Comparison of the distribution of patients with WBRT + boost vs. patients with WBRT only according to prognostic scores (RTOG). Numbers of patients (percentage) are presented, if not otherwise specified. Here, Mann-Whitney U test was used. The reasons for exclusion were as follows: ^1^n = 1 patient with a missing number of brain metastases; ^2^n = 10 patients with CUP, n = 14 patients with primary tumors not considered in the score; ^3^n = 2 patients with CUP, n = 10 patients with a missing number of brain metastases, n = 1 patient with a missing histological subtype in breast cancer, n = 4 patients with primary tumors not considered in the score; ^4^n = 10 patients with CUP, n = 3 patients with missing histology in bronchial carcinoma, n = 5 patients with a missing hemoglobin value, n = 14 patients with primary tumors not considered in the score; ^5^n = 2 patients with CUP, n = 11 patients with a missing number of brain metastases, n = 1 patient with a missing histological subtype in breast cancer, n = 4 patients with primary tumors not considered in the score. WBRT—whole brain radiotherapy. RPA—Recursive Partitioning Analysis. GPA—Graded Prognostic Assessment. Ds-GPA—diagnosis-specific Graded Prognostic Assessment.

| **Parameter** | **WBRT + boost, n = 201** | **WBRT only, n = 52** | **p-value** |
| --- | --- | --- | --- |
| RPA  class 1  class 2  class 3 | 25 (12.4)  147 (73.1)  29 (14.4) | 2 (3.8)  44 (84.6)  6 (11.5) | 0.476 |
| GPA  0 to 1  1.5 to 2  2.5 to 3  3.5 to 4  Not classifiable | 84 (41.8)  82 (40.8)  30 (14.9)  5 (2.5)  0 | 34 (65.4)  14 (26.9)  3 (5.8)  0 (0.0)  1(1.9)^1^ | <0.001 |
| Ds-GPA (2012)  0 to 1  1.5 to 2  2.5 to 3  3.5 to 4  Not classifiable | 63 (31.3)  66 (32.8)  41 (20.4)  7 (3.5)  24 (11.9)^2^ | 17 (32.7)  12 (23.1)  4 (7.7)  2 (3.8)  17 (32.7)^3^ | 0.136 |
| Ds-GPA (updated version)  0 to 1  1.5 to 2  2.5 to 3  3.5 to 4  Not classifiable | 37 (18.4)  64 (31.8)  55 (27.4)  13 (6.5)  32 (15.9)^4^ | 12 (23.1)  15 (28.8)  5 (9.6)  2 (3.8)  18 (34.6)^5^ | 0.029 |
