# Supplementary material for: Radiotherapy for patients with brain metastases and leptomeningeal carcinomatosis: prognostic factors and clinical outcomes
Source: Clin Exp Metastasis. 2025 Jun 2;42(4):33. doi: 10.1007/s10585-025-10352-3 (PMC12130157; doi:10.1007/s10585-025-10352-3)
Supplement: Supplementary file 4 — Supplementary Material 4 [file 10585_2025_10352_MOESM4_ESM.docx]

**Suppl. Table S4:** Comparison of toxicities in patients with WBRT + boost vs. patients with WBRT only. Numbers of patients (percentage) are presented, if not otherwise specified. Only the most frequently occurring side effects are listed here. Neurocognitive and functional outcomes occurred in the following number of patients: concentration impairment n = 2, memory impairment n = 3. ^1^Pearson’s chi-squared test, ^2^Mann-Whitney U test. RT—radiotherapy. WBRT—whole brain radiotherapy.

| **Side effects RT** | **WBRT + boost, n = 201** | **WBRT only, n = 52** | **p-value** |
| --- | --- | --- | --- |
|  |  |  |  |
| Cranial side effects | 77 (38.3) | 16 (30.8) | 0.315^1^ |
| Highest grade |  |  | 0.733^2^ |
| Grade 0 to 1 | 191 (95.0) | 50 (96.2) |  |
| Grade 2 to 4 | 10 (5.0) | 2 (3.8) |  |
| Cephalgia ≥ grade1 | 22 (10.9) | 2 (3.8) | 0.119^1^ |
| Fatigue ≥ grade 1 | 12 (6.0) | 4 (7.7) | 0.649^1^ |
| Alopecia ≥ grade 1 | 21 (10.4) | 0 (0.0) | 0.015^1^ |
| Nausea ≥ grade 1 | 32 (15.9) | 4 (7.7) | 0.130^1^ |
| Leukoencephalopathy | 9 (4.5) | 1 (1.9) | 0.399^1^ |
|  |  |  |  |
