# Supplementary material for: Radiotherapy for patients with brain metastases and leptomeningeal carcinomatosis: prognostic factors and clinical outcomes
Source: Clin Exp Metastasis. 2025 Jun 2;42(4):33. doi: 10.1007/s10585-025-10352-3 (PMC12130157; doi:10.1007/s10585-025-10352-3)
Supplement: Supplementary file 5 — Supplementary Material 5 [file 10585_2025_10352_MOESM5_ESM.docx]

**Suppl. Table S5:** Prognostic factors in patients who received WBRT, univariable Cox regression analysis. ^0^patients who received a cMRI post RT n = 85 patients; unavailable data due to retrospective research at: ^1^n = 13 patients, ^2^n = 12 patients, ^3^n = 12 patients, ^4^n = 4 patients; ^5^n = 156 patients did not receive any systemic therapy concomitant with RT, ^6^n = 71 patients did not receive any systemic therapy 3 months before or after RT. OS—overall survival. ICPFS—intracranial progression-free survival. FFCP—freedom from cranial progression. HR—hazard ratio. CI—confidence interval. RT—radiotherapy. WBRT—whole brain radiotherapy. N.a.—not applicable.

| **Parameter**  **(Number of patients)** | | **OS** | | **ICPFS** | | **FFCP** | | **FFCP^0^** | |
| --- | --- | --- | --- | --- | --- | --- | --- | --- | --- |
|  |  | **HR**  **(95% CI)** | **p-value** | **HR**  **(95% CI)** | **p-value** | **HR**  **(95% CI)** | **p-value** | **HR**  **(95% CI)** | **p-value** |
| Gender  male (143) vs. female (110) | | 1.24  (0.96–1.61) | 0.097 | 1.32  (1.02–1.71) | 0.034 | 1.50  (0.86–2.61) | 0.149 | 1.62  (0.90–2.90) | 0.105 |
| Age at RT | | 1.02  (1.01–1.03) | 0.004 | 1.01  (1.00–1.02) | 0.047 | 0.98  (0.96–1.01) | 0.124 | 1.00  (0.97–1.02) | 0.674 |
| Karnofsky index  ≤median (133) vs. >median (120)  Median: 80 | | 2.50  (1.91–3.27) | <0.001 | 2.06  (1.58–2.67) | <0.001 | 0.72  (0.38–1.37) | 0.322 | 0.78  (0.40–1.55) | 0.482 |
| Charlson Comorbidity index  ≥median (132) vs. <median (121)  Median: 9 | | 1.19  (0.92–1.53) | 0.187 | 1.04  (0.81–1.34) | 0.765 | 0.47  (0.27–0.84) | 0.011 | 0.68  (0.36–1.27) | 0.221 |
| Year of treatment  2016 to 2018 (145) vs.  2019 to 2023 (108) | | 1.11  (0.85–1.43) | 0.448 | 1.10  (0.85–1.43) | 0.465 | 0.96  (0.56–1.67) | 0.896 | 0.99  (0.56–1.77) | 0.980 |
| Number of brain lesions^1^  ≥5 (107) vs. <5 (133) | | 1.43  (1.10–1.86) | 0.008 | 1.48  (1.13–1.92) | 0.004 | 1.53  (0.88–2.69) | 0.135 | 1.65  (0.91–2.99) | 0.096 |
| Primary controlled pre RT  No (48) vs. Yes (205) | | 1.84  (1.34–2.53) | <0.001 | 1.77  (1.28–2.43) | <0.001 | 0.96  (0.41–2.26) | 0.932 | 1.15  (0.45–2.92) | 0.768 |
| Extracranial metastases at brain lesion diagnosis  Yes (148) vs No (105) | | 1.63  (1.25–2.12) | <0.001 | 1.55  (1.19–2.01) | 0.001 | 1.11  (0.64–1.93) | 0.714 | 1.17  (0.66–2.09) | 0.592 |
| Extracranial tumor control pre RT  No (88) vs. Yes (165) | | 1.05  (0.80–1.37) | 0.730 | 1.04  (0.80–1.36) | 0.777 | 1.27  (0.73–2.23) | 0.399 | 1.01  (0.56–1.84) | 0.964 |
| Surgical resection  No (144) vs. Yes (109) | | 1.82  (1.40–2.37) | <0.001 | 1.81  (1.39–2.36) | <0.001 | 1.24  (0.70–2.18) | 0.459 | 1.38  (0.76–2.54) | 0.288 |
| RT side effects  Yes (95) vs. No (158) | | 0.57  (0.44–0.75) | <0.001 | 0.61  (0.47–0.80) | <0.001 | 0.92  (0.53–1.59) | 0.759 | 0.75  (0.42–1.34) | 0.331 |
| RT Boost  Yes (201) vs. No (52) | | 0.71  (0.52–0.98) | 0.036 | 0.78  (0.57–1.08) | 0.129 | 1.70  (0.67–4.28) | 0.264 | 1.79  (0.64–5.00) | 0.266 |
| Total dose applied  ≤30 Gy (68) vs. >30 Gy (185) | | 1.99  (1.49–2.67) | <0.001 | 1.081  (1.36–2.43) | <0.001 | 0.72  (0.31–1.70) | 0.459 | 0.72  (0.26–2.02) | 0.534 |
| Planned total dose  ≤30 Gy (48) vs. >30 Gy (205) | | 1.56  (1.12–2.16) | 0.008 | 1.44  (1.04–1.99) | 0.030 | 0.71  (0.28–1.79) | 0.465 | 0.77  (0.28–2.15) | 0.615 |
| Systemic therapy concomitant with RT  Yes (97) vs. No (156) | | 1.04  (0.80–1.35) | 0.792 | 0.98  (0.76–1.28) | 0.892 | 0.69  (0.38–1.24) | 0.217 | 0.65  (0.35–1.21) | 0.176 |
| Systemic therapy 3 months before or after RT  Yes (182) vs. No (71) | | 0.80  (0.60–1.06) | 0.114 | 0.85  (0.64–1.13) | 0.267 | 1.07  (0.56–2.04) | 0.844 | 1.30  (0.60–2.82) | 0.501 |
| cMRI pre RT  No (19) vs. Yes (234) | | 1.50  (0.93–2.41) | 0.095 | 1.38  (0.86–2.21) | 0.187 | 1.23  (0.38–4.01) | 0.730 | 0.05  (0.00–60932) | 0.673 |
| cMRI post RT  No (168) vs. Yes (85) | | 3.24  (2.44–4.31) | <0.001 | 2.24  (1.70–2.95) | <0.001 | 0.13  (0.05–0.33) | <0.001 | n.a. | n.a. |
| At least 1 lesion in cerebellum^2^  Yes (126) vs. No (115) | | 1.41  (1.08–1.83) | 0.012 | 1.51  (1.16–1.98) | 0.002 | 1.47  (0.84–2.58) | 0.181 | 1.42  (0.78–2.57) | 0.256 |
| At least 1 lesion in brain stem^3^  Yes (27) vs. No (214) | 1.03  (0.69–1.56) | | 0.878 | 1.24  (0.83–1.87) | 0.294 | 1.69  (0.75–3.81) | 0.209 | 1.14  (0.48–2.74) | 0.765 |
| Dexamethasone concomitant with or 3 months post RT  Yes (144) vs. No (109) | 1.26  (0.97–1.63) | | 0.081 | 1.35  (1.04–1.75) | 0.023 | 1.71  (0.97–2.99) | 0.063 | 1.35  (0.75–2.42) | 0.320 |
| Antiepileptics concomitant with or 2 weeks pre or post RT  Yes (58) vs. No (195) | 0.82  (0.60–1.11) | | 0.198 | 0.84  (0.62–1.15) | 0.278 | 1.35  (0.75–2.44) | 0.316 | 1.45  (0.78–2.70) | 0.238 |
| Chemotherapy mono (35) vs. targeted therapy/immune therapy +/− chemotherapy (62) concomitant with RT^5^ | 1.29  (0.85–1.98) | | 0.237 | 1.17  (0.77  (1.79) | 0.466 | 0.69  (0.22–2.15) | 0.526 | 1.13  (0.36–3.58) | 0.830 |
| Chemotherapy mono (72) vs. targeted therapy/immune therapy +/− chemotherapy (110) 3 months before or after RT^6^ | 1.19  (0.88–1.62) | | 0.258 | 1.11  (0.82–1.50) | 0.502 | 0.81  (0.42–1.57) | 0.528 | 1.01  (0.52–1.98) | 0.973 |
| Time between tumor diagnosis and RT^4^ | 0.97  (0.93–1.02) | | 0.234 | 0.99  (0.95–1.03) | 0.602 | 1.00  (0.92–1.08) | 0.941 | 1.07  (0.97–1.19) | 0.173 |
