# Supplementary material for: Radiotherapy for patients with brain metastases and leptomeningeal carcinomatosis: prognostic factors and clinical outcomes
Source: Clin Exp Metastasis. 2025 Jun 2;42(4):33. doi: 10.1007/s10585-025-10352-3 (PMC12130157; doi:10.1007/s10585-025-10352-3)
Supplement: Supplementary file 6 — Supplementary Material 6 [file 10585_2025_10352_MOESM6_ESM.docx]

**Suppl. Table S6:** Prognostic factors in patients who received WBRT, multivariable Cox regression analysis. Endpoint “FFCP”: only “Charlson Comorbidity Index” and “Dexamethasone concomitant with or 3 months post RT” were associated with *p* < 0.05 in univariate analysis. In multivariate analysis, only “Charlson Comorbidity Index ≥ 9” remained significant (HR = 0.52 (95% CI: 0.29-0.91); *p* = 0.023). The effect suggesting that a high CCI is beneficial appears counterintuitive, which may indicate a bias or a spurious association. Therefore, it was not included in the multivariable analysis, and a multivariable model was not conducted at this point. Additionally, “Planned total dose" was not considered for the multivariable model as it showed collinearity with “total dose” (Kendall’s tau b coefficient of 0.81). The variable "cMRI post RT" was highly effected by clinical outcome and thus also omitted from the multivariable model. Unavailable data due to retrospective research at: ^1^n = 13 patients, ^2^n = 12 patients. OS—overall survival. ICPFS—intracranial progression-free survival. FFCP—freedom from cranial progression. HR—hazard ratio. CI—confidence interval. RT—radiotherapy. WBRT—whole brain radiotherapy.

| **Parameter**  **(Number of patients)** | **OS** | | **ICPFS** | |
| --- | --- | --- | --- | --- |
|  | **HR**  **(95% CI)** | **p-value** | **HR**  **(95% CI)** | **p-value** |
| Gender  male (143) vs. female (110) | - | **-** | 1.33  (1.00-1.76) | **0.049** |
| Age at RT | 1.03  (1.01-1.04) | **<0.001** | 1.02  (1.01-1.04) | **0.001** |
| Karnofsky Index  ≤median (133) vs. >median (120)  Median: 80 | 2.31  (1.72-3.10) | **<0.001** | 1.81  (1.36-2.40) | **<0.001** |
| Number of brain lesions^1^  ≥5 (107) vs. <5 (133) | 1.22  (0.87-1.72) | 0.248 | 1.16  (0.82-1.65) | 0.400 |
| Primary controlled pre RT  No (48) vs. Yes (205) | 1.50  (1.06-2.14) | **0.024** | 1.45  (1.01-2.08) | **0.045** |
| Extracranial metastases at brain lesion diagnosis  Yes (148) vs. No (105) | 1.17  (0.87-1.58) | 0.310 | 1.23  (0.92-1.65) | 0.169 |
| Surgical resection  No (144) vs. Yes (109) | 1.38  (1.02-1.86) | 0.038 | 1.21  (0.89-1.64) | 0.226 |
| RT side effects  Yes (95) vs. No (158) | 0.66  (0.49-0.88) | **0.004** | 0.66  (0.50-0.88) | **0.005** |
| RT Boost  No (52) vs. Yes (201) | 3.76  (1.96-7.24) | **<0.001** | - | **-** |
| Total dose applied  ≤30 Gy (68) vs. >30 Gy (185) | 3.94  (2.29-6.80) | **<0.001** | 1.32  (0.91-1.90) | 0.144 |
| At least 1 lesion in cerebellum^2^  Yes (126) vs. No (115) | 1.23  (0.89-1.70) | 0.202 | 1.27  (0.92-1.75) | 0.148 |
| Steroids concomitant with or 3 months post RT  Yes (174) vs. No (136) | - | **-** | 1.55  (1.16-2.06) | **0.003** |
