# Supplementary material for: Radiotherapy for patients with brain metastases and leptomeningeal carcinomatosis: prognostic factors and clinical outcomes
Source: Clin Exp Metastasis. 2025 Jun 2;42(4):33. doi: 10.1007/s10585-025-10352-3 (PMC12130157; doi:10.1007/s10585-025-10352-3)
Supplement: Supplementary file 7 — Supplementary Material 7 [file 10585_2025_10352_MOESM7_ESM.docx]

**Suppl. Table S7:** Prognostic factors in overall study collective, univariable Cox regression analysis. ^0^patients who received a cMRI post RT n = 109 patients; unavailable data due to retrospective research at: ^1^n = 15 patients, ^2^n = 14 patients, ^3^n = 14 patients, ^4^n = 4 patients; ^5^n = 187 patients did not receive any systemic therapy concomitant with RT, ^6^n = 85 patients did not receive any systemic therapy 3 months before or after RT; OS—overall survival. ICPFS—intracranial progression-free survival. FFCP—freedom from cranial progression. HR—hazard ratio. CI—confidence interval. RT—radiotherapy. WBRT—whole brain radiotherapy. N.a.—not applicable.

| **Parameter**  **(Number of patients)** | | **OS** | | **ICPFS** | | **FFCP** | | **FFCP^0^** | |
| --- | --- | --- | --- | --- | --- | --- | --- | --- | --- |
|  |  | **HR**  **(95% CI)** | **p-value** | **HR**  **(95% CI)** | **p-value** | **HR**  **(95% CI)** | **p-value** | **HR**  **(95% CI)** | **p-value** |
| Gender  male (173) vs. female (137) | | 1.18  (0.93–1.49) | 0.167 | 1.22  (0.97–1.54) | 0.089 | 1.38  (0.86–2.22) | 0.183 | 1.40  (0.85–2.32) | 0.189 |
| Age at RT | | 1.02  (1.01–1.03) | 0.001 | 1.01  (1.00–1.02) | 0.064 | 0.98  (0.97–1.00) | 0.059 | 0.99  (0.98–1.01) | 0.505 |
| Karnofsky Index  ≤median (159) vs. >median (151)  Median: 80 | | 2.36  (1.85–3.00) | <0.001 | 1.86  (1.47–2.35) | <0.001 | 0.76  (0.45–1.30) | 0.320 | 0.80  (0.45–1.44) | 0.463 |
| Charlson Comorbidity Index  ≥median (157) vs. <median (153)  Median: 9 | | 1.20  (0.95–1.51) | 0.129 | 0.99  (0.78–1.24) | 0.907 | 0.47  (0.29–0.78) | 0.003 | 0.69  (0.40–1.19) | 0.180 |
| Year of treatment  2016 to 2018 (163) vs.  2019 to 2023 (147) | | 1.17  (0.93–1.48) | 0.189 | 1.13  (0.90–1.42) | 0.300 | 0.96  (0.60–1.54) | 0.875 | 1.00  (0.61–1.64) | 0.991 |
| Number of brain lesions^1^  ≥5 (126) vs. <5 (169) | | 1.45  (1.14–1.84) | 0.003 | 1.55  (1.22–1.98) | <0.001 | 1.39  (0.85–2.28) | 0.192 | 1.39  (0.83–2.34) | 0.217 |
| Primary controlled pre RT  No (59) vs. Yes (251) | | 1.82  (1.36–2.42) | <0.001 | 1.75  (1.31–2.34) | <0.001 | 0.96  (0.46–2.02) | 0.921 | 1.09  (0.50–2.41) | 0.823 |
| Extracranial metastases at brain lesion diagnosis  Yes (180) vs. No (130) | | 1.60  (1.26–2.04) | <0.001 | 1.42  (1.12–1.80) | 0.004 | 0.96  (0.60–1.55) | 0.881 | 0.91  (0.55–1.50) | 0.705 |
| Extracranial tumor control pre RT  No (108) vs. Yes (202) | | 1.03  (0.81–1.31) | 0.826 | 1.10  (0.86–1.40) | 0.462 | 1.37  (0.85–2.22) | 0.200 | 1.08  (0.64–1.80) | 0.782 |
| Leptomeningeal carcinomatosis  Yes (35) vs. No (275) | | 1.54  (1.07–2.20) | 0.020 | 1.39  (0.97–1.98) | 0.072 | 1.19  (0.54–2.60) | 0.671 | 1.08  (0.43–2.70) | 0.874 |
| Surgical resection  No (177) vs. Yes (133) | | 1.79  (1.41–2.26) | <0.001 | 1.92  (1.51–2.44) | <0.001 | 1.28  (0.79–2.07) | 0.324 | 1.33  (0.79–2.22) | 0.278 |
|  | |  |  |  |  |  |  |  |  |
| RT side effects  Yes (122) vs. No (188) | | 0.58  (0.45–0.73) | <0.001 | 0.66  (0.52–0.84) | 0.001 | 0.98  (0.61–1.57) | 0.935 | 0.91  (0.55–1.49) | 0.698 |
| WBRT  Yes (287) vs. No (23) | | 2.13  (1.32–3.45) | 0.002 | 1.54  (0.97–2.46) | 0.068 | 0.62  (0.33–1.19) | 0.151 | 0.75  (0.39–1.45) | 0.399 |
| Total dose applied  ≤30 Gy (109) vs. >30 Gy (201) | | 1.75  (1.37–2.23) | <0.001 | 1.76  (1.38–2.24) | <0.001 | 1.07  (0.61–1.87) | 0.827 | 1.15  (0.63–2.09) | 0.647 |
| Planned total dose  ≤30 Gy (84) vs. >30 Gy (226) | | 1.26  (0.97–1.63) | 0.081 | 1.27  (0.98–1.64) | 0.076 | 0.91  (0.50–1.63) | 0.740 | 1.08  (0.59–2.00) | 0.797 |
| Systemic therapy concomitant with RT  Yes (123) vs. No (187) | | 0.94  (0.74–1.19) | 0.604 | 0.97  (0.77–1.23) | 0.824 | 0.99  (0.61–1.59) | 0.950 | 0.92  (0.55–1.52) | 0.741 |
| Systemic therapy 3 months before or after RT  Yes (225) vs. No (85) | | 0.77  (0.59–0.99) | 0.044 | 0.87  (0.67–1.13) | 0.296 | 1.19  (0.67–2.12) | 0.552 | 1.25  (0.65–2.41) | 0.509 |
| cMRI pre RT  No (20) vs. Yes (290) | | 1.58  (1.00–2.50) | 0.051 | 1.42  (0.90–2.24) | 0.136 | 1.06  (0.33–3.41) | 0.921 | 0.05  (0.00–24,055.72) | 0.652 |
| cMRI post RT  No (201) vs. Yes (109) | | 3.37  (2.60–4.35) | <0.001 | 2.25  (1.75–2.87) | <0.001 | 0.14  (0.07–0.32) | <0.001 | n.a. | n.a. |
| At least 1 lesion in cerebellum^2^  Yes (153) vs. No (143) | | 1.34  (1.06–1.70) | 0.016 | 1.55  (1.22–1.98) | <0.001 | 1.67  (1.03–2.72) | 0.038 | 1.60  (0.95–2.68) | 0.075 |
| At least 1 lesion in brain stem^3^  Yes (32) vs, No (264) | 1.09  (0.75–1.58) | | 0.660 | 1.20  (0.83–1.74) | 0.333 | 1.15  (0.53–2.54) | 0.721 | 0.83  (0.36–1.94) | 0.671 |
| Steroids concomitant with or 3 months post RT  Yes (174) vs. No (136) | 1.34  (1.06–1.69) | | 0.016 | 1.46  (1.15–1.84) | 0.002 | 1.49  (0.92–2.40) | 0.102 | 1.37  (0.83–2.27) | 0.213 |
| Antiepileptics concomitant with or 2 weeks pre or post RT  Yes (68) vs. No (242) | 0.77  (0.58–1.02) | | 0.069 | 0.81  (0.61–1.08) | 0.148 | 1.30  (0.78–2.17) | 0.316 | 1.42  (0.83–2.44) | 0.205 |
| Chemotherapy mono (41) vs. targeted therapy/immune therapy +/− chemotherapy (82) concomitant with RT^5^ | 1.36  (0.92–2.00) | | 0.123 | 1.21  (0.82–1.77) | 0.334 | 0.80  (0.34–1.89) | 0.615 | 1.11  (0.44–2.78) | 0.831 |
| Chemotherapy mono (84) vs. targeted therapy/immune therapy +/− chemotherapy (141) 3 months before or after RT^6^ | 1.39  (1.05–1.83) | | 0.022 | 1.19  (0.90–1.57) | 0.228 | 0.81  (0.45–1.45) | 0.479 | 0.98  (0.54–1.80) | 0.952 |
| Time between tumor diagnosis and RT^4^ | 0.98  (0.95–1.02) | | 0.327 | 1.00  (0.96–1.03) | 0.859 | 1.03  (0.98–1.09) | 0.274 | 1.11  (1.03–1.19) | 0.008 |
